# Supplementary material for: Mapping the evolution of fertility support policies in China: A content and instrumental analysis
Source: PLoS One. 2025 Oct 9;20(10):e0332137. doi: 10.1371/journal.pone.0332137 (PMC12510515; doi:10.1371/journal.pone.0332137)
Supplement: S1 Appendix — (ZIP) [file pone.0332137.s001.zip › S1 Appendix. 226 original policy documents/136-中共中央、国务院关于实施全面两孩政策改革完善计划生育服务管理的决定(FBM-CLI-16-262093).doc]

中共中央、国务院关于实施全面两孩政策改革完善计划生育服务管理的决定

**发布部门：** [中国共产党中央委员会](https://www.pkulaw.com/law/chl?Aggs.IssueDepartment=a01) [国务院](https://www.pkulaw.com/law/chl?Aggs.IssueDepartment=5)

**发布日期：**2015.12.31

**实施日期：**2015.12.31

**时效性：** [现行有效](https://www.pkulaw.com/law/chl?Aggs.TimelinessDic=01)

**效力级别：** [党内法规制度](https://www.pkulaw.com/law/chl?Aggs.EffectivenessDic=XR12)

**法规类别：** [计划生育管理](https://www.pkulaw.com/law/chl?Aggs.Category=09705)

中共中央、国务院关于实施全面两孩政策 改革完善计划生育服务管理的决定
（2015年12月31日）

　　实行计划生育是我国的一项基本国策，事关亿万家庭的幸福安康，事关全面建成小康社会和“两个一百年”奋斗目标的实现。为贯彻落实党的十八大和十八届二中、三中、四中、五中全会精神，适应人口和经济社会发展新形势，促进人口长期均衡发展，现就全面实施一对夫妇可生育两个孩子政策（以下简称全面两孩政策），进一步改革完善计划生育服务管理，作出如下决定。
　　**一、**充分认识实施全面两孩政策、改革完善计划生育服务管理的重大意义
　　（一）计划生育工作取得了举世瞩目的伟大成就。20世纪五六十年代，随着经济恢复、社会安定、人民生活改善和医疗卫生保障水平的提高，我国总人口从新中国成立初期的5.4亿人迅速增加到1970年的8.3亿人，给经济社会发展带来了巨大压力。为控制人口过快增长，国家从七十年代开始在城乡推行计划生育。1980年，党中央发表《关于控制我国人口增长问题致全体共产党员、共青团员的公开信》，提倡一对夫妇生育一个孩子。1982年，计划生育被确定为基本国策。40多年来，我国实施人口与发展综合决策，不断完善计划生育政策，走出了一条中国特色统筹解决人口问题的道路。人口过快增长的势头得到有效控制，资源、环境压力有效缓解，妇女儿童发展状况极大改善，人口素质明显提高，促进了经济快速发展和社会进步，有力支撑了改革开放和社会主义现代化事业，为全面建成小康社会奠定了坚实基础。我国实行计划生育也为世界人口发展和减贫作出了重大贡献，树立了负责任大国的良好形象。在此过程中，亿万人民群众积极响应党和国家号召，自觉实行计划生育，作出了巨大贡献；广大人口和计划生育工作者付出了心血和汗水。实践证明，实行计划生育符合我国国情，是正确的，得到了广大人民群众的理解和支持，是强国富民安天下的伟大事业。
　　（二）我国人口发展呈现出重大转折性变化。人口问题始终是人类社会共同面对的基础性、全局性和战略性问题。人口的趋势性变化，将对经济社会发展产生全面、深刻、长远的影响。进入21世纪特别是“十二五”时期以来，我国人口发展的内在动力和外部条件发生了显著变化。人口总量增长势头明显减弱，劳动年龄人口和育龄妇女开始减少，老龄化程度不断加深；群众生育观念发生重大转变，少生优生已成为社会生育观念的主流；家庭规模趋向小型化，养老抚幼功能弱化；人口红利减弱，以人力资本为核心的国际竞争优势有待进一步加强。这些变化对人口安全和经济社会发展带来新的挑战。同时还应清醒地看到，到本世纪中叶，我国人口总量仍将保持在13亿以上，人口众多的基本国情不会根本改变，人口对经济社会发展的压力不会根本改变，人口与资源环境的紧张关系不会根本改变。当前，我国正处于人口大国向人力资本强国转变的关键时期。必须从全局和战略高度出发，充分认识坚持计划生育基本国策的重要性和长期性，立足国情，遵循规律，正确处理当前与长远、总量与结构、人口与资源环境的关系，逐步调整完善生育政策，促进人口长期均衡发展，最大限度发挥人口对经济社会发展的能动作用，牢牢把握战略主动权。
　　（三）实施全面两孩政策、改革完善计划生育服务管理是新形势下坚持计划生育基本国策的重大战略部署。党的十八届三中全会作出了坚持计划生育基本国策、启动实施单独两孩政策的重大决策。单独两孩政策稳妥扎实有序实施，为进一步调整完善生育政策积累了经验，当前启动实施全面两孩政策条件具备、时机成熟。实施全面两孩政策、改革完善计划生育服务管理，是促进人口长期均衡发展的重大举措，有利于优化人口结构，增加劳动力供给，减缓人口老龄化压力；有利于促进经济社会持续健康发展，实现全面建成小康社会的奋斗目标；有利于更好地落实计划生育基本国策，促进家庭幸福与社会和谐。各级党委和政府要充分认识实施全面两孩政策、改革完善计划生育服务管理的重要性，增强责任感和使命感，用法治的思维、创新的精神和务实的作风，不断探索新形势下落实计划生育基本国策的体制机制和方式方法，使计划生育成为惠及亿万家庭的甜蜜事业。

　　**二、**指导思想、基本原则和主要目标
　　（四）指导思想
　　高举中国特色社会主义伟大旗帜，全面贯彻党的十八大和十八届二中、三中、四中、五中全会精神，以邓小平理论、“三个代表”重要思想、科学发展观为指导，深入贯彻习近平总书记系列重要讲话精神，坚持“四个全面”战略布局，以增进家庭和谐幸福、促进人口长期均衡发展为主线，坚持计划生育基本国策，统筹推进生育政策、服务管理制度、家庭发展支持体系和治理机制综合改革，努力实现规模适度、素质较高、结构优化、分布合理的人口均衡发展，促进人口与经济社会、资源环境协调可持续发展，为实现中华民族伟大复兴的中国梦提供坚实基础和持久动力。
　　（五）基本原则
　　--以人为本。尊重家庭在计划生育中的主体地位，坚持权利与义务对等，寓管理于服务之中，引导群众负责任、有计划地生育。
　　--创新发展。推动人口和计划生育工作由控制人口数量为主向调控总量、提升素质和优化结构并举转变，由管理为主向更加注重服务家庭转变，由主要依靠政府力量向政府、社会和公民多元共治转变。
　　--法治引领。充分发挥立法对完善生育政策和服务管理改革的引领、规范、保障作用，坚持严格、规范、公正、文明执法，不断提高计划生育法治水平。
　　--统筹推进。注重改革措施的系统性、整体性、协同性，做到调整完善生育政策与服务管理改革同步推进、配套政策措施同步制定。
　　（六）主要目标
　　到2020年，计划生育服务管理制度和家庭发展支持体系较为完善，政府依法履行职责、社会广泛参与、群众诚信自律的多元共治格局基本形成，计划生育治理能力全面提高；覆盖城乡、布局合理、功能完备、便捷高效的妇幼保健计划生育服务体系更加完善，基本实现人人享有计划生育优质服务，推动联合国2030年可持续发展议程的落实；保持适度生育水平，人口总量控制在规划目标之内。

　　**三、**稳妥扎实有序实施全面两孩政策
　　（七）依法组织实施全面两孩政策。贯彻落实新修改的《[中华人民共和国人口与计划生育法](https://www.pkulaw.com/chl/18c6c388fc14eaa9bdfb.html)》，完善相关行政法规以及地方性法规。从2016年开始实施全面两孩政策。各省（自治区、直辖市）政府综合评估本地人口发展形势、计划生育工作基础和政策实施风险，科学制定实施方案，报国务院主管部门备案，确保政策平稳落地，生育水平不出现大幅波动。
　　（八）改革生育服务管理制度。实行生育登记服务制度，对生育两个以内（含两个）孩子的，不实行审批，由家庭自主安排生育。改进再婚等情形再生育管理。优化办事流程，简化办理手续，全面推行网上办事，进一步简政便民。依法依规查处政策外多孩生育。
　　（九）加强出生人口监测预测。加强人口变动情况调查，科学预测出生人口变动趋势，建立出生人口监测和预警机制。加快推进国家人口基础信息库和人口健康信息化建设，实现国家与省级人口和计划生育信息互联互通，实现户籍管理、婚姻、人口健康、教育、社会保障等信息共享。
　　（十）合理配置公共服务资源。根据生育服务需求和人口变动情况，合理配置妇幼保健、儿童照料、学前和中小学教育、社会保障等资源，满足新增公共服务需求。引导和鼓励社会力量举办非营利性妇女儿童医院、普惠性托儿所和幼儿园等服务机构。

　　**四、**大力提升计划生育服务管理水平
　　（十一）加强妇幼健康计划生育服务。推进优生优育全程服务，落实孕前优生健康检查，加强孕产期保健服务和出生缺陷综合防治，提高出生人口素质。向不孕不育等生育困难人员提供必要的辅助生殖技术服务。推进妇幼保健计划生育服务机构标准化建设和规范化管理，加强孕产妇与新生儿危急重症救治能力建设。加快产科和儿科医师、助产士及护士人才培养，合理确定服务价格，在薪酬分配等方面加大政策倾斜力度。全面推进知情选择，向育龄人群提供安全、有效、适宜的避孕节育服务，提高服务的公平性和可及性。加强基础研究和科技创新，开发推广避孕节育、优生优育、生殖保健的新技术新产品。
　　（十二）推进流动人口基本公共卫生计生服务均等化。按照常住人口配置服务资源，将流动人口纳入城镇基本公共卫生和计划生育服务范围。巩固完善流动人口信息互通、服务互补、管理互动的全国“一盘棋”工作机制。推进网上信息核查和共享，做好流动人口在居住地的生育登记服务。广泛开展生殖健康科普宣传，增强流动人口等人群自我保健意识和防护能力。关怀关爱流动人口和留守人群，促进社会融合。
　　（十三）强化基层基础工作。完善宣传倡导、依法管理、优质服务、政策推动、综合治理的计划生育长效工作机制。深入开展计划生育优质服务先进单位创建活动。建立健全卫生和计划生育综合监督行政执法体系，加强计划生育服务管理能力建设。稳定和加强县、乡级计划生育工作力量，妥善解决好村级计划生育专干的报酬待遇、养老保障等问题。
　　（十四）充分发挥社会组织作用。加强政府与社会协同治理，广泛动员工会、共青团、妇联等群团组织和其他社会组织共同做好计划生育工作。充分发挥计划生育协会的生力军作用，切实加强县、乡级计划生育协会的组织和能力建设，更好地承担宣传教育、生殖健康咨询服务、优生优育指导、计划生育家庭帮扶、权益维护和流动人口服务等工作。鼓励社会组织依法开展人口和计划生育公益慈善与帮扶救助活动。在城乡社区和企事业单位，引导群众广泛开展计划生育自我管理、自我服务、自我教育、自我监督。

　　**五、**构建有利于计划生育的家庭发展支持体系
　　（十五）加大对计划生育家庭扶助力度。切实保障计划生育家庭合法权益，使他们优先分享改革发展的成果。对政策调整前的独生子女家庭和农村计划生育双女家庭，继续实行现行各项奖励扶助政策，在社会保障、集体收益分配、就业创业、新农村建设等方面予以倾斜。完善计划生育家庭奖励扶助制度和特别扶助制度，实行扶助标准动态调整。帮扶存在特殊困难的计划生育家庭，妥善解决他们的生活照料、养老保障、大病治疗和精神慰藉等问题。推进计划生育与扶贫开发相结合，继续实施“少生快富”工程。对政策调整后自愿只生育一个子女的夫妻，不再实行独生子女父母奖励优惠等政策。
　　（十六）增强家庭抚幼和养老功能。建立完善包括生育支持、幼儿养育、青少年发展、老人赡养、病残照料等在内的家庭发展政策，鼓励按政策生育。完善计划生育奖励假制度。增强社区幼儿照料、托老日间照料和居家养老等服务功能。推进医疗卫生与养老服务相结合，探索建立长期护理保险制度。加大对残疾人家庭、贫困家庭和独居老人的帮扶支持力度。广泛开展创建幸福家庭活动和新家庭计划。
　　（十七）促进社会性别平等。深入开展关爱女孩行动，创造有利于女孩成长成才的社会环境。综合治理出生人口性别比偏高问题，依法严厉打击非医学需要的胎儿性别鉴定和选择性别人工终止妊娠行为。依法保障妇女的宅基地、房屋等财产继承权和土地承包权。依法保障女性就业、休假等合法权益，支持女性生育后重返工作岗位，鼓励用人单位制定有利于职工平衡工作与家庭关系的措施。

　　**六、**切实加强组织领导
　　（十八）落实党政责任。各级党委和政府要坚持计划生育党政一把手亲自抓、负总责，将实施全面两孩政策、改革完善计划生育服务管理作为全面深化改革的重要任务，加强统筹规划、政策协调和工作落实。坚持和完善计划生育目标管理责任制，确保责任到位、措施到位、投入到位。对人口和计划生育工作主要目标任务未完成、严重弄虚作假、违法行政造成恶劣影响等情形，实行“一票否决”。
　　（十九）加强部门协作。进一步完善计划生育兼职委员和领导小组制度。建立健全重大经济社会政策人口发展影响评估机制，促进相关经济社会政策与计划生育政策有效衔接。各有关部门要认真履行职责，重点解决好政策配套、公共服务保障、执法协调、信息互通等问题，加强对各地工作的指导。
　　（二十）深化人口发展战略研究。深入研究新形势下人口与经济、社会、资源、环境之间的互动关系以及人口数量、素质、结构和分布的变动趋势，加强前瞻性研究，完善人口发展战略，编制中长期规划。科学评估经济增长和社会发展对生育行为的影响，准确研判生育水平变动态势，做好政策储备。加强人口发展的国际比较研究，促进国际交流与合作。
　　（二十一）做好宣传和舆论引导。各地区各部门要把思想和行动统一到中央的决策部署上来，大力宣传计划生育取得的伟大成就，做好实施全面两孩政策解读。加强人口基本国情和计划生育基本国策教育，不断增强全社会的国情和国策意识。总结推广计划生育服务管理改革的好经验、好做法，表彰先进典型。正确引导社会舆论，营造支持政策落实和改革创新的良好氛围。
　　（二十二）强化督导落实。各地区各有关部门要按照本决定要求，制定实施方案，细化改革任务，明确实施步骤。加强对各项改革措施的跟踪评估，及时发现和解决改革中的苗头性、倾向性问题。各省（自治区、直辖市）党委和政府每年要向中央专题报告本地区计划生育工作情况，中央将定期开展督查。

© 北大法宝：（[www.pkulaw.com](https://www.pkulaw.com/)) 专业提供法律信息、法学知识和法律软件领域各类解决方案。北大法宝为您提供丰富的参考资料，正式引用法规条文时请与标准文本核对。
欢迎查看所有[产品和服务](https://www.pkulaw.com/helps/2.html)。法宝快讯：[如何快速找到您需要的检索结果？](https://www.pkulaw.com/helps/70.html)[法宝](https://www.pkulaw.com/helps/69.html)V6有何新特色？

扫描二维码阅读原文

**原文链接：**<https://www.pkulaw.com/chl/8564756bae805261bdfb.html>
